# Stress Incontinence during Different High-Impact Exercises in Women: A Pilot Survey

**DOI:** 10.3390/ijerph17228372

**Published:** 2020-11-12

**Authors:** Iman Akef Khowailed, Joelle Pinjuv-Turney, Cathy Lu, Haneul Lee

**Affiliations:** 1 College of Rehabilitative Sciences, University of St Augustine for Health Sciences, San Marcos, CA 92069, USA; 2 School of Physical Therapy, Touro University Nevada, Henderson, NV 89014, USA; Dpt18.joelle.pinjuv-turney@nv.touro.edu (J.P.-T.); Dpt18.cathy.lu@nv.touro.edu (C.L.); 3 Department of Physical Therapy, Gachon University, Incheon 21936, Korea

**Keywords:** stress urinary incontinence, incontinence, high-intensity exercise, parity, pelvic floor, women’s health

## Abstract

The aim of this survey was to investigate the prevalence of stress urinary incontinence (SUI) among women (primigravida, multigravida, and nulligravida) in high-impact exercise groups—CrossFit, kickboxing, and boot camp. Incontinence Survey was modified to an anonymous online questionnaire. A total of 17 participants, 64.2% reported at least some urinary leakage during exercise. About 85.7% of participants in each of the 3 high-intensity exercise groups exercised >3 h/week. There was no significant difference in the likelihood of urinary leakage between participants who have had at least 1 pregnancy and those who had never been pregnant. CrossFit group were significantly more likely to report urinary leakage than those in the kickboxing and boot camp groups combined (*p* = 0.023). The participants did not exhibit typical risk factors, as they were premenopausal, active, and had an average body mass index within the normal range. This pilot survey indicates that all women those who do high-impact exercises are susceptible to stress urinary incontinence (SUI), and that CrossFit poses a greater risk for SUI in terms of more jumping resulting in increased intra-abdominal pressure and ground reaction forces compared to others. Our pilot study indicates that a higher risk of SUI during high impact exercises may exist associated with previous pregnancy but also in nulliparous women.

## 1. Introduction

Urinary incontinence (UI) is a common condition that inconveniences persons of all ages and sexes. Approximately 200 million people worldwide are affected by UI [1]. UI is a general term used to describe the involuntary leakage of urine; however, UI can be differentiated into 3 common types: stress, urgency, and mixed incontinence [2]. Stress urinary incontinence (SUI) is the most common type of UI and defined as urinary leakage upon exertion, such as during coughing, sneezing, and physical activity [2].

Most studies reported the prevalence of UI among women in the general population to range from 25% to 45% [3]. Among women aged between 15 to 64 years, prevalence rates ranging from 10% to 55% have been reported [4]. Among them, SUI is the most common type of UI, and the majority of the 13 million affected Americans are women [5]. Physical activity, especially high-impact exercise, has become popular in recent years among the general population, and more people are now training at the elite athlete level. High-impact training, while beneficial to physical and mental health, can also be an independent risk factor for SUI. Strenuous physical activity is interesting in that it may have a positive effect on the pelvic floor by strengthening it, but it may also have a negative effect by weakening the pelvic floor muscles (PFMs) because of overload. SUI may negatively affect the lives of physically active women and cause them to alter or cease their level of performance.

Women may be more susceptible to SUI due to PFM dysfunction. Persons who are unable to contract their PFMs may strain or contract other muscles such as the hip adductors, abdominals, and gluteus muscles [4]. These muscles differ from PFMs in that they cannot provide structural support for the pelvic organs or prevent bladder descent in the presence of increased abdominal pressure [4]. According to Bø, elite female athletes experience sudden increases in intra-abdominal pressure (IAP) that can cause SUI owing to the lack of, weak, or delayed co-contraction of the PFMs and other muscle groups. The PFMs of female athletes may be conditioned for normal day-to-day activities; however, with increased pressure on the PFMs during exercise, urine leakage may occur during such activities [4].

Other common established risk factors for SUI in women include pregnancy and childbirth. Parity, particularly giving birth via vaginal delivery, is relevant because pregnancy and childbirth by themselves may cause structural changes and nerve damage that result in urinary leakage [1]. In the study by Rortveit et al., the prevalence of UI was 10.1% among nulliparous women, 15.9% in women who delivered via cesarean section, and 21% in women who delivered via vaginal delivery [6]. This may be due to overstretching and weakening of the PFMs and connective tissue from the stress of pregnancy. Rortveit et al. also found that women who delivered via cesarean section had an increased risk of both stress and mixed incontinence, whereas women who delivered vaginally had an increased risk of only SUI [6]. Laxity of the pelvic floor can also interfere with the contractibility of the striated urethral sphincter against increased IAP or contractions of the detrusor muscle [3]. The literature has shown that a dysfunctional pelvic floor and high-impact physical activity contribute to SUI before pregnancy, whereas low-impact activity promotes continence [7]. Fozzatti et al. compared nulliparous women who attended gyms and performed high-impact exercises with women who did not exercise regularly and found that the former group had a higher prevalence of UI symptoms [8]. Lindland et al. investigated young nulliparous women with SUI and found that strenuous exercise—consisting of a 90-min interval training program—resulted in lower maximum voluntary vaginal contraction pressure, which indicated PFM fatigue [9].

High-impact exercises are defined as exercises that involve lower-extremity weight bearing and/or exercises in which both feet are off the ground at the same time with abrupt repeated increase in IAP [8]. Goldstick and Constantini found that activities that involve jumping tend to induce the most urinary leakage [10]. Hay concluded that the peak vertical ground reaction forces during high-impact activities can range up to 14.0–22.3 times the body weight [11]. In addition to collecting data about training and urinary symptoms of female ball players compared with non-athletes, Borin and colleagues measured their intracavity perineal pressure with a perineometer during perineal isometric contractions [12]. While ground reaction forces cannot directly affect the pelvic floor, the researchers correlated ground reaction forces with decreased perineal pressure, as they found that lower perineal pressures increased symptoms of SUI. Moreover, their results indicated that female athletes had lower perineal pressures [12].

Urine loss in athletes is related to how frequently the athletes are subjected to increased intra-abdominal pressure, which is caused by a contraction of the abdominal muscles in high-impact activities without proper awareness of the perineal muscles. Strenuous physical activity that involves intra-abdominal pressure can overload and chronically damage the perineum [4], thus decreasing the contraction force of pelvic floor muscles (PFM) and increasing the risk for SUI. The more frequent the impact associated with increased intra-abdominal pressure, the greater the need for restraint and support by the PFM, which must be strengthened to preserve their function and prevent SUI [4].

One possible hypothesis of the association between UI and high-impact exercise is that intra-abdominal pressure rises dramatically during strenuous physical activity to the point that it exceeds the intra-urethral pressure. When the intra-abdominal pressure increases during physical exertion, such as exercise, coughing, or sneezing, the natural mechanism of urinary continence is a mechanical compression of the urethral sphincter against the supporting endopelvic fascia of the vagina [13]. In addition, Nygaard et al. [14] suggested that hypothalamic amenorrhea that results from intense exercise and eating disorders lowers estrogen levels, which also would contribute to the onset of UI.

The purpose of this study is to evaluate the incidence of SUI in physically active women, and to examine specific high impact exercises that may increase the incidence of SUI in 3 distinct high-impact exercise groups—CrossFit, kickboxing, and boot camp. These exercises contain jumping, hopping, and leaping components were chosen because they are recently some of the most popular fitness workouts among women.

## 2. Materials and Methods

### 2.1. Ethical Approval

All protocols and procedures were approved by Touro University Nevada Institute Review Board (IRB9-13-17C) for an expedite review and all protocols and procedures were explained to each subject prior to their participation in this study.

### 2.2. Participants

A total of 20 women who are attending kickboxing, CrossFit, or boot camp classes at least 3 times per week aged between 18 and 40 participated in the anonymous survey. Percipients were excluded with the following criteria: at menopausal, currently pregnant, any past medical history of diabetes, hypertension, cardiovascular diseases, metabolic diseases, hysterectomy, uterine cancer, or pelvic floor prolapse. Subjects were included if they reported any leaks during each specific exercise ranging and could range from rarely, sometimes, often, and always.

Of these, 3 responses were excluded (2 responses from respondents who were outside the age range of 18–40 years and 1 response from a respondent who reported being pregnant). Therefore, a total of 17 respondents were eligible, including 3 who did not complete the survey.

### 2.3. Questionnaire

A modified questionnaire was used from the Female Athlete Survey: Urinary Incontinence Survey [15]. The study identified the prevalence of SUI among female athletes who participated in high-impact sports. It also included specific questions aimed at identifying SUI symptoms during high-impact activities [15]. We did not include the question concerning the participants’ willingness to try exercises for their urinary leakage because we were not assessing the need for preventative UI education. We decided to retain the questions regarding the participants’ willingness to seek treatment for their leakage and their general knowledge about preventative exercises to assess their awareness of SUI. It was important for us to keep these questions because the responses to them may encourage medical professionals to educate their patients about pelvic floor health.

We adapted Carls’ survey to design a more comprehensive survey to better suit our inclusion and exclusion criteria. We specifically selected 3 high-impact exercises to identify and compare the exercises that had the highest and lowest prevalence of associated SUI symptoms. Choosing common and popular high-intensity exercise classes provided us a large population from which to recruit participants. In addition, we further asked questions about the number of pregnancies each participant has had, and separated them into the primigravida, multigravida, and nulligravida groups. Additional questions about the participants’ susceptibility to SUI involved smoking history, body mass index (BMI), oral contraceptive use, mode of childbirth delivery, perineal trauma, and bowel/bladder dysfunction. We included a nulligravida group because we wanted an unbiased data set of women without the typical risk factors of SUI, to allow us to identify the common high-intensity exercises that pose the biggest risk for SUI.

We included questions that were aimed to exclude participants who were not eligible for this study. Our exclusion criteria were created to prevent biases from factors associated with SUI, such as menopause, current pregnancy, and comorbidities. We decided to leave the 2 questions about urge tendencies to help separate participants experiencing urgency incontinence and those with stress incontinence.

With consent from the owners and managers of gym facilities, we sent our research flyers containing the link to the survey to Camp Rhino, Xtreme Couture mixed martial arts school, and CrossFit Modulus (gyms located in Las Vegas, Nevada that offer the specific exercise classes we were interested in).

We searched for local CrossFit, kickboxing, and boot camp Facebook pages. We asked the most popular pages for permission to post our flyer to their page and/or send out our flyer to their members and clients. We messaged 8 CrossFit, 7 kickboxing, and 8 boot camp Facebook pages.

The survey responses were anonymous. The survey was completed electronically and privately, and discretion was ensured owing to the sensitive and personal nature of the survey questions. Interested and eligible participants took the online survey, which included a consent form and questions to screen for eligibility.

### 2.4. Statistical Analysis

Survey data were summarized by computing descriptive statistics, and Fisher’s exact tests were used to test independence between variables of interest, when sample sizes supported such tests. Analyses were done in R 3.4.1 software (R Core Team, Vienna, Austria).

## 3. Results

Among 17 eligible respondents, three did not complete the survey so total of 14 respondents were included for statistical analysis. In our study, 10 participants reported no pregnancies, 4 participants had given birth via vaginal delivery, 2 participants had given birth via cesarean section, and 1 participant had given birth via both modes.

**Figure 1:**
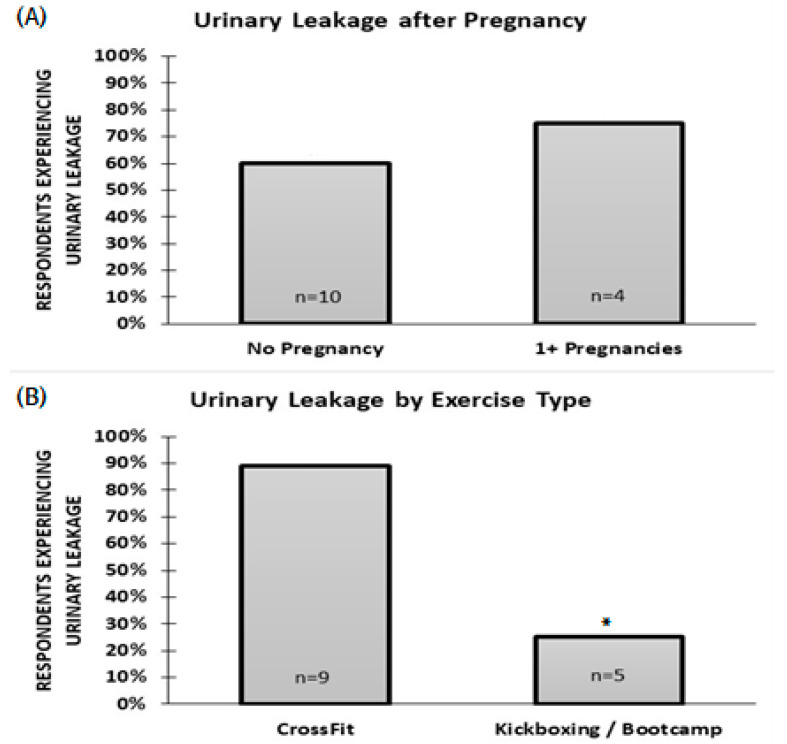
(**A**): Percent of respondents experiencing urinary leakage based on past pregnancy. (**B**): Percent of respondents experiencing urinary leakage based on exercise type. * statistical significant of the of urinary leakage between exercise type; Fisher’s exact test.

A total of 64.2% of the respondents reported experiencing at least some leakage, and 85.7% exercised more than three hours per week (Table 1). The likelihood of leakage in those who have had at least 1 pregnancy was not different from those who were never pregnant (*p* > 0.05; Figure 1). However, leakage was more likely in those who attend CrossFit classes than in those who attend kickboxing or boot camp classes (*p* = 0.023; Figure 1).

Most respondents reported small amounts of leakage that occurs on a recurring but infrequent weekly and yearly basis (Table 2). Jumping and intra-abdominal contraction associated with coughing, sneezing, or laughing triggered urinary leakage in 78% of the respondents, and running or sports triggered leakage in 67% (Table 3). Only 33% of the respondents reported leakage triggered by psychological cues or lifting activities. Few respondents reported that urinary leakage interfered with their lives; 1 of 9 reported that urinary leakage had interfered with hobbies, and 2 of 9 reported that it had interfered with sports or exercise.

## 4. Discussions

High-impact exercise increases the IAP with certain activities such as jumping, burpees, high kicks, and box jumps. Increased IAP and ground reaction forces to the pelvic floor have been shown to increase the likelihood of SUI among parous women. Although our study had a small sample size of 17 participants, our results indicate that SUI is prevalent in young healthy women those who do high-impact exercises associated with previous pregnancy but also in nulliparous physically active women. This study may contribute to many women not seeking treatment for their urinary leakage owing to lack of awareness.

All women in our study were found to be equally likely to have urinary leakage regardless of the number of pregnancies. Previous study reported that most women with SUI who engage in physical activity and sports are nulliparous, younger, and have a lower BMI, which are all consistent with our study findings [16]. Our participants did not exhibit typical risk factors for SUI, as they were premenopausal, active, and had an average BMI that was within the normal range. Therefore, we concluded that all women are susceptible to SUI.

Our results were also similar to those observed in a cross-sectional survey investigating UI among women after multiple pregnancies found no significant correlation between age and gravidity with SUI [17]. However, there is also evidence in the literature suggesting a positive correlation between childbirth and urinary leakage. A meta-analysis conducted by Tähtinen et al. found that vaginal delivery increased the risk of developing urinary leakage with exertion by nearly 2-fold compared with cesarean section [18]. Rortveit et al. reported that the odds of SUI risk among cesarean section deliveries were 1.6 times the odds of no deliveries, those among vaginal deliveries were 3.0 times the odds of no deliveries, and those among vaginal deliveries were 2.4 times the odds of cesarean section deliveries [6].

The triggers for leakage were divided into 5 groups: intra-abdominal (coughing, sneezing, laughing), lifting (weight lifting, lifting, squatting), jumping (double under, jump, jump rope, box jumps), running/sports (exercise, sport, running), and psychological cues (walking to the bathroom, hearing running water). Psychological cue triggers were indications of urgency incontinence. We grouped weight lifting and squats into a single group, as these exercises constituted the smallest triggers for leakage. We believe this is because lifting exercises do not require both feet to leave the ground simultaneously. Our results showed that coughing, sneezing, laughing, and jumping exercises are the most frequent triggers of leakage, and we attributed this finding to the high increase in IAP during these tasks. Increases in IAP cause a caudodorsal displacement of the urethra and a damaged or weak pelvic floor support provides less resistance to this stretch and affects the closure of the urethral lumen, which then results in incontinence [19]. We conclude that coughing and sneezing induce the same amount of IAP as jumping activities. Thus, women who do not participate in high-impact exercises are equally likely to be at a risk for SUI.

Our study found that women who participate in CrossFit were significantly more likely to have urinary leakage than those who participate in kickboxing or boot camp. Eliasson et al. found that 80% of the nulliparous elite trampolinists in their study had involuntary leakage during jumping training, as confirmed with a pad test [7]. We suspect that this correlates with our findings because CrossFit involves more jumping than kickboxing and boot camp. Similarly, Middlekauff et al. found that 27.7% of their participants who engage in an average of 4.27 ± 0.96 days per week of CrossFit workouts had symptoms of urinary leakage related to activity, coughing, or sneezing compared with only 8.57% of participants who do not engage in strenuous activities [20].

The pathophysiology of SUI with high impact exercises are possibly related to sudden increases in intra-abdominal pressure that can lead to a weakened elastic recoil and neuromuscular fatigability over time [21]. The main hypotheses governing the literature explaining how improper physical training affects the pelvic floor muscles [4] and possibly contributes to SUI. The “hammock hypothesis” functions on the belief that physical activity weakens pelvic floor muscles due to the stretch of connective tissues from constant force [22,23]. This hypothesis states that increased intra-abdominal pressure stretches the ligaments and fascial tissues of pelvic floor muscles leading to permanent damage of the tissue [24,25]. Increased intra-abdominal pressure is thought to fatigue muscles of the pelvic floor as they balance forces. Therefore, when the downward force from the abdomen is not balanced by the upward force from the pelvic floor muscles, stress urinary continence can occur [26]. This is similar to the mechanism explaining how childbirth damages pelvic floor muscles, and therefore this process is often thought to be the cause of SUI among nulliparous females [24]. The hammock hypothesis suggests that urinary symptoms, such as SUI, occur when the increase in abdominal pressure exceeds the threshold of the urinary sphincter [27,28]. This hypothesis also supports beliefs that SUI is related to muscle fatigue, evidenced by more frequent leakage later in the day and with more exercises [26].

Previous research hypothesized that increased IAP in female athletes causes their PFM to endure increased overloading and stretching during strenuous activities such as high-impact exercise [4]. While the other study reported no significant difference in PFM strength before and after exercise between participants who engage in CrossFit and those who do not engage in strenuous activities [20]. We believe that high-impact exercises do not specifically target the PFMs; therefore, all athletes are at a high risk for SUI. None of the respondents had sought treatment for SUI. Our participants reported infrequent urinary leakage within the past week and year. As the reported quantity of leakage ranged from small to minimal, none of our participants required the use of pads. We hypothesized that none of them sought treatment because of the low frequency of leakage episodes and the small amount of leakage. Most of the respondents were familiar with Kegel exercises. Since the participants were not asked whether they performed Kegel exercises, it is unknown if they actively contracted their PFMs to strengthen those muscles. On the basis of our results and those of related literature on this topic, we believe that women experience urinary leakage not only because of intense exercise but because of improper training of their PFMs.

The limitations of this study were largely due to the small sample size (17 participants) for data analysis. The modes of childbirth were combined to compare women who have had at least 1 pregnancy and those who had never been pregnant. Future research should include a larger sample size and should identify the leakage triggers during specific exercises. Different types of jumping exercises should be compared, and their relationship to the severity of SUI, due to the high ground reaction forces generated during jumping, should be investigated. Since we hypothesized that all women are susceptible to SUI, women of all activity levels should be studied to determine any correlation between exercise intensity and SUI, as our study only targeted women engaging in high-intensity exercises. Next, this study is at risk of selection bias, as only those interested in the topic, because of pelvic floor symptoms, could have answered the survey call. Further researcher should use probability sampling methods to reduce a selection bias. Lack of pelvic floor education, the belief that leakage during high-impact exercise is normal, and embarrassment over leakage are other factors that could contribute to women not seeking treatment for UI. Therefore, more research concerning pelvic floor dysfunction and UI are warranted to educate athletes, strength coaches, personal trainers, women’s health specialists, and practitioners about the risk. Research regarding the PFM among the athletic population are needed to help educate the health and fitness community about the importance of targeted strength training of the PFMs during functional activities. Such education will help increase women’s health awareness, and this may address the negative stigma related to pelvic floor disorders and dysfunctions.

## 5. Conclusion

In conclusion, most of the women enrolled in this pilot study reported symptoms of UI whether they are primigravida, multigravida, or nulligravida. Healthy physically active women who practice high-impact exercises should be aware that a higher risk of SUI may exist. Our results may provide helpful recommendations to women participating in high-impact exercises.

## Figures and Tables

**Table 1:** Descriptive information on survey respondents.

	N (%)	Weight (kg)	Height (cm)	BMI (kg/m^2^)
Overall	14 (100.0)	64.1 ± 8.7	166.9 ± 4.9	23.0 ± 2.6
1+ completed pregnancies	4 (28.6)	63.6 ± 9.7	163.9 ± 6.4	23.6 ± 2.4
No completed pregnancies	10 (71.4)	64.3 ± 8.8	168.2 ± 3.9	22.7 ± 2.8
CrossFit	9 (64.2)	60.2 ± 5.7	163.9 ± 4.0	22.4 ± 2.3
Kickboxing or Bootcamp	5 (35.8)	71.8 ± 8.0	171.8 ± 3.4	24.5 ± 2.7
No leakage	5 (35.8)	68.1 ± 7.1	170.2 ± 5.1	23.5 ± 1.4
Leakage	9 (64.2)	62.5 ± 9.0	164.5 ± 4.0	23.1 ± 3.2
<3 h exercise per week	2 (14.3)	72.4 ± 0.9	172.7 ± 0.0	24.3 ± 0.4
3–6 h exercise per week	9 (64.2)	62.8 ± 10.0	166.5 ± 5.3	22.6 ± 3.2
>6 h exercise per week	3 (21.5)	62.3 ± 3.4	164.3 ± 1.4	23.1 ± 1.3

Abbreviation: BMI, Body mass index.

**Table 2:** Urinary incontinence episode frequency and amount of urine leakage reported by survey respondents.

Leakage Frequency Past Week ^1^		Leakage Frequency Past Year ^2^		Leakage Amount ^3^	
Never	5	None	5	None	5
Once	0	Daily	0	A few drops	6
Rarely	2	2–4 × / week	1	Small amount	3
Sometimes	7	Weekly	1	Soak a pad	0
Frequently	0	2–4 × / month	2	Floods	0
Every Session	0	Monthly	5		

^1^ frequency of episodes of urinary incontinence in the past week; ^2^ frequency of episodes of urinary incontinence in the past year; ^3^ amount of urine leakage per episode.

**Table 3:** Activities that have triggered urinary incontinence.

Situation of Urinary Incontinence	N (%) ^1^
Intra-abdominal (cough, sneeze, laugh)	7 (77.8)
Lifting (weight lifting, lifting, squat clear)	3 (33.3)
Jumping (2× under, jump, jump rope, box jumps)	7 (77.8)
Running/Sports (exercise, sport, running)	6 (66.7)
Psychological cue (walking to bathroom, water running)	3 (33.3)

^1^ number provided answers out of nine respondents who reported any leakage.
